# Using Single-Particle Inductively Coupled Plasma Mass Spectrometry to Determine the Changes of Silver Nanoparticles in Bread Induced via Simulated Digestion

**DOI:** 10.3390/foods13091311

**Published:** 2024-04-24

**Authors:** Diomid Revenco, Martina Fialová Hakenová, Oto Mestek, Richard Koplík

**Affiliations:** 1 Department of Food Analysis and Nutrition, University of Chemistry and Technology, Prague 6, 166 28 Prague, Czech Republic; diomid1.revenco@vscht.cz (D.R.); martina.fialova25@seznam.cz (M.F.H.); 2 Department of Analytical Chemistry, University of Chemistry and Technology, Prague 6, 166 28 Prague, Czech Republic; oto.mestek@vscht.cz

**Keywords:** silver nanoparticles, sp-ICP-MS, simulated digestion, food safety, bread, food contamination

## Abstract

Silver nanoparticles (AgNPs), widely used in various fields of technology as an antimicrobial agent, represent a new type of environmental pollutant. Through various routes, AgNPs might penetrate into agricultural crops and foodstuffs. It is important to know if AgNPs contained in food persist in digested food and are therefore available for entering the inner organs of the consumer’s body. Using the technique of single-particle ICP-MS, we analysed the changes in the number and size distribution of AgNPs added to a sample of bread submitted to in vitro simulated gastrointestinal digestion. The majority of silver, in terms of mass, was transformed from the state of particles to the dissolved state during bread digestion, but the number of particles was reduced by 25% only. The most abundant particle size was reduced from 60 nm to 49 nm. Hence, a substantial part of transformed nanoparticles is still present in food digestate. This means that AgNPs consumed together with food can theoretically enter the inner cells of human body.

## 1. Introduction

The development in nanotechnologies and the increasing use of nanomaterials in various fields of industry, technology, and agriculture bring not only benefits but also potential risks. Commonly used nanomaterials include inorganic nanoparticles such as silver nanoparticles (AgNPs), gold nanoparticles (AuNPs), metal oxide nanoparticles (e.g., ZnONPs or TiO_2_NPs), platinum-group-metal nanoparticles (e.g., PdNPs) etc. [1,2]. AgNPs are mainly used for their antimicrobial effects as excipients in textile products, cosmetics, and as components of composite materials in packaging technology [3,4]. The worldwide production of AgNPs has already reached the level of hundreds of tonnes per year [5]. The use and re-use of the products containing AgNPs therefore necessarily introduces silver, in a nanoparticulate form, into the environment; mainly via contaminated wastewater. Consequently, AgNPs may enter the bodies of aquatic biota and, through other routes, they may also penetrate into non-aquatic ecosystems [6].

The content of nanoparticulate matter in a liquid sample, such as some natural water, is expressed as particle number concentration *N*; i.e., the number of particles dispersed in unitary sample volume. The common unit of *N* is mL^−1^ or the thousand times larger unit L^−1^. In addition to particle number concentration, it is necessary to know the size (diameter) and elemental composition of the nanoparticles. 

All of these data are accessible via analysis performed using single-particle inductively coupled plasma mass spectrometry (sp-ICP-MS). There are, of course, other instrumental techniques of nanoparticle analysis, such as transmission electron microscopy with energy-dispersive X-ray spectroscopy, dynamic light scattering, field-flow fractionation hyphenated with ICP-MS or ICP-OES, and UV–Vis spectroscopy [7]. In contrast to electron microscope techniques of nanoparticle analysis that are excellent in detection of both larger and very small NP (with a diameter of several nm or even smaller), the technique of sp-ICP-MS stands out in its ability to determine metallic nanoparticles quantitatively even if they are present at very low particle number concentrations.

The analytical technique of sp-ICP-MS was developed in the early years of the 21st century and has been refined and optimised over the past decade for the determination of nanoparticles of various elements. The basics are described, for example, in the papers [8,9,10,11]. As far as the application of sp-ICP-MS in food analysis is concerned, this technique was recently used for the determination of AgNPs in ground beef [12], for the determination of TiO_2_NPs in various foods [13], for the stability tests of ZnONPs released from food-packaging materials [14], and for the determination of a wider range of inorganic nanoparticles (Al_2_O_3_NPs, SiO_2_NPs, TiO_2_NPs, Cr_2_O_3_NPs, Fe_2_O_3_NPs, CuONPs, ZnONPs) in various foodstuffs [15].

In contrast to the conventional ICP-MS that is used to analyse samples in the form of true solutions (mostly aqueous solutions acidified with HNO_3_), in sp ICP-MS, the analysed sample is an aqueous dispersion of solid particles of varying sizes (in the range of tens of nanometers to units of micrometers). Due to very high measurement frequency (or very short dwell time period) applied in single-particle analysis, each individual particle introduced into the spectrometer gives a sharp and very short signal of analyte intensity (i.e., a peak of width of *ca* 0.5 ms). During analysis, which takes one or two minutes, the spectrometer records several thousand such peaks, or even more. Each peak is integrated and then, the whole set of recorded peaks is classified according to the size (integral intensity or peak area *A*_NP_). Particle number concentration *N* is then calculated from the number of recorded peaks *X* according to Equation (1):

(1) $$N=\frac{60\cdot X}{t\cdot \dot{V}\cdot \eta }\;[{\mathrm{mL}}^{-1}],$$

in which *t* is total duration of measurement [s], $\dot{V}$ is sample aspiration flow rate [mL min^−1^] and *η* is transport efficiency (dimensionless quantity). 

Using the result of classification of peaks according to the peak area, the distribution of particle sizes can be calculated. Firstly, the mass of the element in a single particle is derived from the peak area on the basis of measurement calibration, which utilises standard dispersion of nanoparticles of the known mean diameter (and therefore, the known mean mass), according to Equation (2):

(2) $$m_{\mathrm{NP}}=\frac{m_{M}}{w}=\frac{A_{\mathrm{NP}}}{k\cdot w}\;[\mathrm{fg}],$$

in which *m*_M_ [fg] is the mass of the detected element in the nanoparticle, *w* is the mass fraction of the element in the material of nanoparticle (*w* = 1 for nanoparticles made of a pure metal), *A*_NP_ is the peak area [count] and *k* [count fg^−1^] is the slope of the calibration straight line for the dependence of the recorded peak area on the mass of the particle.

Secondly, the diameter of a particle, *d*_NP_, expressed in nanometers, is derived from the particle mass using a presumption of spherical shape of particles according to Equation (3):

(3) $$d_{\mathrm{NP}}=\sqrt[{3}]{\frac{6\times 10^{6}\cdot m_{\mathrm{NP}}}{\pi \cdot \rho }}\;[\mathrm{nm}],$$

in which *ρ* is the density of the nanoparticle material [g cm^−3^].

The distribution of the masses or diameters of the particles in a sample population is normally shown as a histogram (frequency versus mass or diameter).

As the AgNPs are massively used in various common products, the contamination of the food chain and crude materials used for food production by silver nanoparticles cannot be ruled out. Silver in the form of ions is of low toxicity and the total amounts of silver in food are very low when expressed in terms of mass (on the order of ng g^−1^ [5]). However, even trace amounts of silver (expressed in terms of mass) represent a considerable number of particles when silver is present in nanoparticle form. For example, the mass of a 100 nm spherical silver particle is 5.493 fg, while the number of silver atoms in one such particle is approximately 30,664,000. This means that 1 ng of silver in the form of these nanoparticles will contain approx. 182,000 particles. 

In the case of AgNPs, we cannot be sure of their harmlessness. Due to the size of the nanoparticles, it is possible that they penetrate cells. A nanoparticle inside a cell is a foreign body. Therefore, the contamination of food with AgNPs could theoretically pose a risk of damage to the cells of the consumer’s body [16].

These considerations necessarily lead to the question of AgNPs stability in food during food digestion in the human gastrointestinal tract. Publications devoted to this question are still rather scarce. However, these papers suggest that AgNPs exposed to digestive enzymes persist, at least in part, in the sample dispersion [16].

In this paper, we tested the effect of simulated digestion on the changes AgNPs added to the sample of wheat bread. The experiment design was set in a form that enables the control of possible errors of sp-ICP-MS analysis caused by the sample matrix.

## 2. Materials and Methods

### 2.1. Standards, Chemicals, and Samples

Standards of AgNPs of NanoXact grade were purchased from nanoComposix, San Diego, CA, USA. The standards are provided as dispersion in citrate buffer solution. The producer claims that mass concentration of silver in the dispersions is 20 µg mL^−1^. The guaranteed diameters of nanoparticles in individual standard dispersions are 18.6 ± 2.7 nm, 40 ± 4 nm, 59 ± 5 nm and 97 ± 11 nm. 

Standard solutions of elements Ag and Rh were purchased from Analytika, s.r.o., Prague, Czech Republic. The mass concentrations were 1000 mg L^−1^ in the matrix of 2% HNO_3_.

Other chemicals used in experiments were nitric acid, 65%, Suprapur^®^ grade (Merck, Darmstadt, Germany), hydrochloric acid, 30%, Suprapur^®^ grade (Merck, Darmstadt, Germany), ammonium bicarbonate purissimum grade (Fluka, Seelze, Germany), pepsin from porcine gastric mucosa (Sigma-Aldrich, Burlington, MA, USA), pancreatin from porcine pancreas (Sigma-Aldrich, Burlington, MA, USA), and gelatin from porcine skin (Sigma-Aldrich, Burlington, MA, USA).

Demineralised water (resistivity of 18.2 MΩ cm^−1^) was used for the preparation of all solutions and dispersions. It was prepared by distillation and demineralisation by Milli-Q (Millipore, Bedford, MA, USA).

As a food sample, white wheat bread was used. The bread was purchased in common food market (Billa, spol. s.r.o., Modletice, Czech Republic). Only the inner part of the loaf was taken as a sample.

### 2.2. Analytical Instrument

Sp ICP-MS and conventional ICP-MS analyses were carried out on a NexION 350D (Perkin Elmer, Norwalk, CT, USA) instrument equipped with an argon-saving torch, a glass cyclonic spray chamber, and a glass concentric nebuliser. The instrument was controlled by Syngystix software (version 1.1). For all ICP-MS analyses, the instrument operated under the following conditions: RF power 1100 W, argon flows 11 L min^−1^ and 1.0 L min^−1^ (for plasma and auxiliary flow, respectively), detector voltage (pulse stage) 800 V, rejection parameters 0 and 0.25 (for RPa and RPq, respectively). The variable voltage of quadrupole ion deflector was applied. The spray chamber was cooled to 2 °C. Instrumental conditions of analysis are summarised in Table 1.

### 2.3. Characterisation of AgNPs Standard Dispersions

There are two main parameters characterising standard dispersion of AgNPs: the particle number concentration and the most frequent particle diameter. The producer declares average particle diameter with the standard deviation and total mass concentration of Ag (20 µg mL^−1^). The more precise estimation of particle number concentration in standards is accessible via exact silver mass concentration and the particles’ diameter distribution. The former can be found by a conventional quantitative analysis using ICP-MS, while the latter results from a sp ICP-MS analysis. 

The exact mass concentration of silver in stock AgNPs dispersions was determined by conventional ICP-MS method. The dispersions were first decomposed in microwave decomposition unit with high-purity nitric acid and the resulting solutions were diluted to receive the expected silver mass concentration of 10 ng mL^−1^, whereas the diluted Rh solution was added as internal standard to receive the final Rh concentration of 20 ng mL^−1^. Silver was quantified using standard solutions (5, 10 and 20 ng mL^−1^) prepared from stock silver solution that contained also Rh at the same level. The found mass concentrations of silver in stock standards of AgNPs were 21.47 (*s* = 0.16) and 23.79 (*s* = 0.03) µg mL^−1^ for 59 nm and 97 nm AgNPs standards, respectively.

The diluted dispersions of these AgNPs standards were then analysed by sp-ICP-MS method to determine the size distribution of the nanoparticles. The expected values of particle number concentration of the standard dispersion calculated from the declared average diameters and from the declared silver mass concentration were 1.77 × 10^10^ mL^−1^ and 3.99 × 10^9^ mL^−1^ for 59 nm and 97 nm standards, respectively. The diluted dispersions were prepared in the matrix of gelatin aqueous solution (0.5 g L^−1^) to receive particle number concentration of approx. 10^5^ mL^−1^. The results were obtained as the table showing the number of found particles *x*_d_ of the specific diameter *d* (in nm) against diameter *d*. The step of diameter was 1 nm, so the range of diameter was 15 to 100 nm and 15 to 150 nm, respectively. The values of particle mass of specific diameter *m*_d_ given in fg were then calculated for each diameter category by expressing *m*_NP_ from Equation (3) on the presumption that the silver density *ρ* = 10.49 g cm^−3^ and silver mass fraction *w* = 1. Then, the total mass of silver contained in the recorded particles was calculated as a sum of products:

(4) $$m_{\mathrm{total}}=\sum_{d}m_{d}\cdot x_{d}\;[\mathrm{fg}]$$

The total number of particles recorded during sp-ICP-MS analysis *X* is the sum of *x*_d_ values:

(5) $$X=\sum_{d}x_{d}$$

The average mass of a particle in the population of particles present in the standard dispersion is given as the ratio of total silver mass in recorded particles and the number of recorded particles:

(6) $${\bar{m}}_{\mathrm{particle}}=m_{\mathrm{total}}/X\;[\mathrm{fg}].$$

Now, the particle number concentration *N* of AgNPs can be calculated from exact mass concentration (expressed in fg mL^−1^), determined by ICP-MS, and the average particle mass (expressed in fg):

(7) $$N=c_{\mathrm{Ag}}/{\bar{m}}_{\mathrm{particle}}\;[{\mathrm{mL}}^{-1}].$$

The found *N* values are given in Table 2.

### 2.4. Sample Preparation 

Three variants of digestates were prepared: sample digestates (A), blank digestates (B), and control digestates (C). The preparation of blank digestates (B) was performed without any portion of the food matrix; this means that the sole AgNPs (of the average size of 59 nm) were treated. The sample digestates (A) contained both AgNPs (59 nm) and the food sample since the start of procedure, whereas the control digestates (C) contained only food sample matrix from the start and were spiked with AgNPs prior to measurement (i.e., at the final dilution step). The check measurements were also performed with the diluted standard dispersion of 59 nm AgNPs (S) and double blank (BB).

The in vitro digestion was performed in two steps simulating gastric and intestinal periods of the real physiological process. In the next paragraph, the procedure used for sample A is described. The differences in preparation of B, C, S and BB are apparent from the experiment design outlined in Table 3 below. 

To prepare the sample A, a portion of 0.5 of bread (only a mass of crumbs) was weighed into 60 mL polypropylene screw-capped bottle and exactly 1 mL of ten-times diluted standard dispersion of AgNPs (*d* = 59 ± 5 nm, *N* = 1.70 × 10^9^ mL^−1^, *c*_Ag_ = 2.147 µg mL^−1^) was added. Then, 40 mL of hydrochloric acid solution (0.02 mol L^−1^) and 25 mg of pepsin were added. The bottle was kept in a thermostat at 37 °C for 3 h and constantly shaken using a horizontal laboratory shaker. The sample was cooled down to 20 °C and pH was adjusted to 7.5 by dropwise addition of ammonium bicarbonate solution (1 mol L^−1^). Then, 50 mg of pancreatin was added and the sample was again incubated for 3 h in the same conditions. The digestate of the appearance of fine suspension was cooled down to 20 °C and quantitatively transferred into 100 mL volumetric flask, diluted to the mark with ultrapure water, mixed and centrifuged for 5 min at 200× *g*. The supernatant was used for final sample dilution just before sp-ICP-MS analysis. To dilute the sample, 0.25 mL of the supernatant was pipetted into 50 mL volumetric flask, 2.5 mL of 1% aqueous solution of gelatin was added, and the flask was made up to the mark with ultrapure water.

Sample B was prepared in an analogous way as sample A, yet without the food matrix.

Control sample C was prepared using a procedure analogous to sample A, nevertheless, without addition of AgNPs at the beginning. Instead of this, the nanoparticles were added to the digestate of bread at the moment of final dilution. To a 50 mL volumetric flask, the following liquids were pipetted: 0.25 mL of the digestate supernatant, 2.5 mL of gelatin solution and 0.5 mL of 2000-times diluted stock standard dispersion of AgNPs (*d* = 59 ± 5 nm, *N* = 0.85 × 10^7^ mL^−1^, *c*_Ag_ = 10.735 ng mL^−1^). 

Samples A, B and C were prepared in a such a way to achieve the same final concentration of silver (0.107 ng mL^−1^) and the same expected particle number concentration of AgNPs of 85,000 mL^−1^. The same concentration of silver and AgNPs was also in the standard (S) used to compare the results of analyses of samples A, B, C with the original unchanged state of the system. The differences among preparation of standard (S) and samples A, B, C and double blank are summarised in Table 3 that illustrates the experiment design.

### 2.5. Single-Particle ICP-MS Analysis

In principle, the analysis of an unknown aqueous sample containing metal nanoparticles consists of the several steps:

1. Optimisation of main instrument conditions to achieve the best sensitivity in conventional analysis mode and setting of sample aspiration rate to optimum (in accordance with suggested dwell time setting), see Loula et al. [17] for details;
2. Determination of the exact value of sample aspiration flow rate (weighing of aspirated water) and inserting the found value as the parameter necessary for result calculation provided by software, for details, see [17];
3. Determination of transport efficiency in particle analysis mode; this is achieved via measurement of standard dispersion of nanoparticles of known value of *N*;
4. Calibration peak area vs. nanoparticles mass relation using several standard dispersions of nanoparticles of known diameters or even using single standard dispersion mentioned in step 3;
5. Measurement of samples (the samples must be sufficiently diluted in order to avoid interferences and errors in measurements [17,18]);
6. Data evaluation and results calculation.

As outlined above, before switching to single-particle analysis mode, the instrument was optimised at operating in the standard mode of solution analysis. Namely, torch alignment and nebuliser gas flow optimisation routines were applied to achieve maximum sensitivity for ^107^Ag detection when 1 ng mL^−1^ solution of Ag in 1% HNO_3_ was nebulised. After optimisation, the spray chamber was rinsed by 10 min nebulisation of 1% HNO_3_ and 10 min nebulisation of ultrapure water. Then, the instrument was set to single-particle analysis operation mode (see Table 1) and the exact value of sample aspiration flow rate was determined by repeated weighing of aspirated water. The found exact value of flow rate was entered into software parameters. 

In the next step, the transport efficiency was determined using standard dispersion of AgNPs of the diameter 59 nm in diluted gelatin solution (*N* = 85,000 mL^−1^). This dispersion is identical with standard S described in Section 2.5. The software calculated transport efficiency value (in principle, via expressing *η* from Equation (1)). The value of *η* fluctuated day to day, ranging from 0.03 to 0.04.

**Figure 1:**
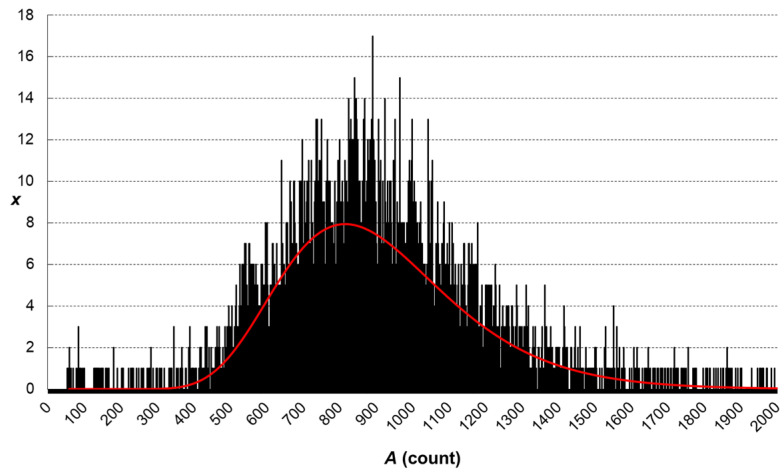
Example of frequency-peak area distribution recorded for standard dispersion of AgNPs of the nominal diameter of 97 nm (the red line is fitting curve given by software assuming log-normal distribution).

Then, the calibration of particle size was performed. It consisted of the analyses of four diluted standard dispersions of AgNPs of the nominal diameters of 18.6, 40, 59 and 97 nm prepared in a diluted gelatin solution. The individual stock standard dispersions were diluted to achieve the particle number concentration of approx. 10^5^ mL^−1^ in the final dispersion nebulised into the instrument (*N*_18.6_ = 0.71 × 10^5^ mL^−1^, *N*_40_ = 1.14 × 10^5^ mL^−1^, *N*_59_ = 0.85 × 10^5^ mL^−1^ and *N*_97_ = 0.998 × 10^5^ mL^−1^). The masses corresponding to above-mentioned nominal diameters of AgNPs are 0.0353, 0.3515, 1.1281 and 5.0129 fg, respectively. To receive calibration line (peak area vs. particle mass), the found peak areas corresponding to fitted maximum of recorded frequency-area distribution (see example in Figure 1) were plotted against nominal particle mass. Peak area vs. particle mass plot is a straight line, while the peak area vs. particle diameter is a cubic curve.

When the calibration procedures were performed, the analyses of individual test dispersions (standard S, samples A, B, C and double blank BB) were conducted. For each test dispersion, a minimum of four measurements were performed. The recorded data and corresponding results calculated by Syngystix software were exported to Microsoft Excel (version 14.0.7268.5000, 2010) file for the final data evaluation. The whole analysis starting from sample preparation was repeated five times.

## 3. Results

The behaviour of inorganic nanoparticles in the various matrixes of foods is under ongoing research [4,19,20]. It is important, because some foods might be contaminated with nanomaterials. As far as silver is concerned, its total amount naturally present in most foods (except mushrooms and buckwheat) is very low (<0.2–1.5 ng g^−1^) [20,21].

To assess the potential hazard of AgNPs present in food, it is necessary to comprehend their behaviour during digestion of food and estimate their possible absorption. Therefore, we performed in vitro digestion of wheat breadcrumbs samples spiked with AgNPs and analysed the treated samples using the sp ICP-MS technique. Owing to its advantageous narrow range in size, we selected the standard AgNPs of the nominal diameter of 59 nm. All test dispersions intended for immediate single-particle analysis were stabilised by gelatin, as this agent was proven to be effective in stabilisation of AgNPs in aqueous dispersions [17]. We simulated the gastric and intestinal steps of digestion, without emulsification of the fats to keep the procedure as simple as possible. The behaviour of AgNPs is affected by their contact with the digested food matrix and reagents (enzymes) as well as by changing the pH value over the course of in vitro testing.

**Figure 2:**
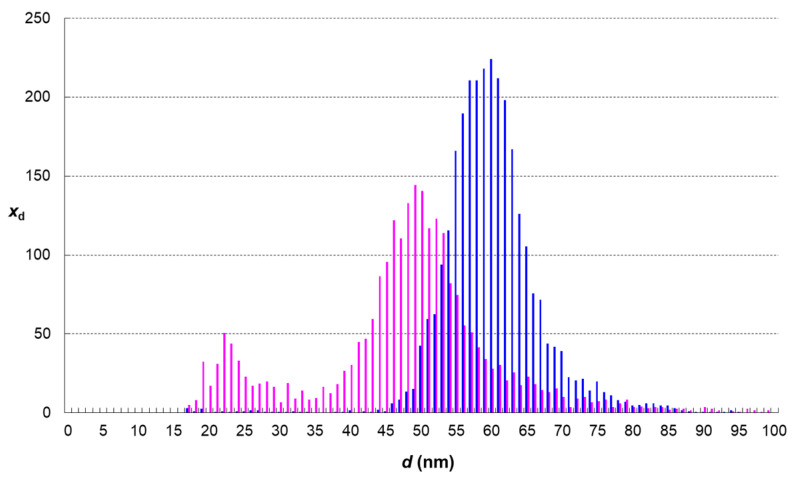
Change of particle size distribution in the dispersion of AgNPs that have undergone simulated digestion together with wheat bread (pink histogram—sample A, blue histogram—standard S).

**Figure 3:**
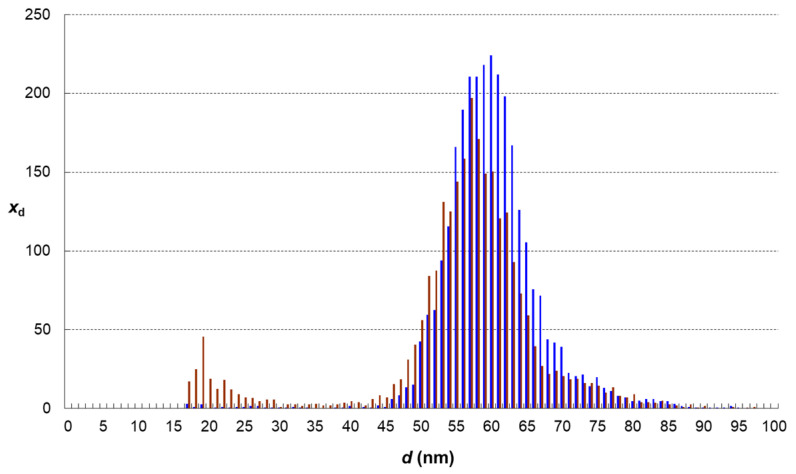
Change of particle size distribution in the dispersion of AgNPs that have undergone simulated digestion without food matrix (brown histogram—sample B, blue histogram—standard S).

**Figure 4:**
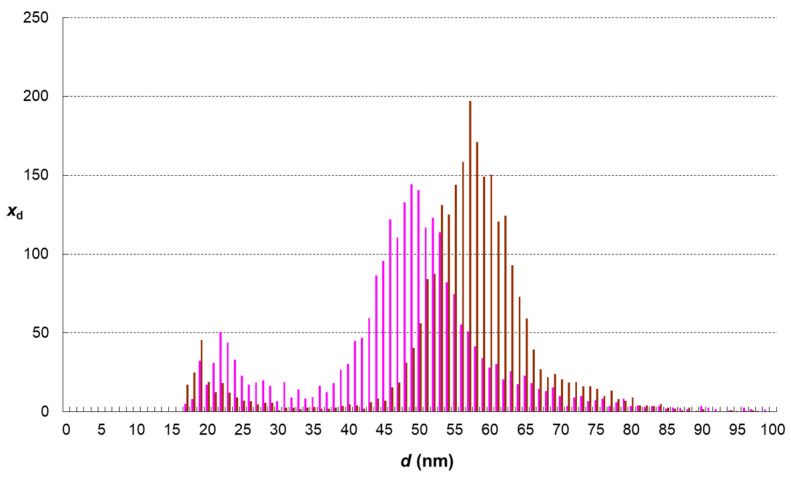
Comparison of AgNPs size distribution in samples digested with (pink histogram—sample A) and without bread matrix (brown histogram—sample B).

**Figure 5:**
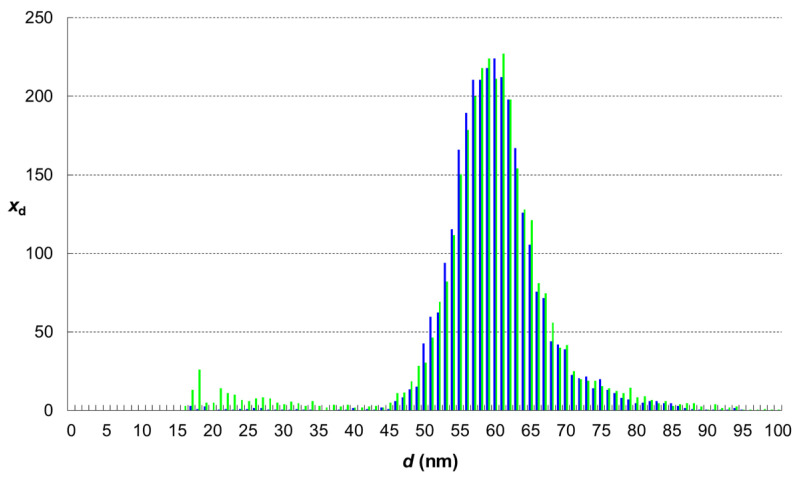
Comparison of AgNPs size distribution in control sample (green histogram—sample C) and intact dispersion of nanoparticles (blue histogram—standard S).

The results of single-particle analyses of AgNPs displayed in following figures (Figure 2, Figure 3, Figure 4 and Figure 5) represent average distributions determined from five independent sets of digestions and analyses.

The analysis of sample A reveals particle size distribution changes induced by the process of food digestion performed together with AgNPs. This distribution is displayed on Figure 2 together with that of the unchanged nanoparticle standard. A noticeable reduction in size was observed. The most abundant diameter was reduced from 60 nm to 49 nm, while a minority of smaller nanoparticles with a diameter of about 22 nm appeared in the sample. Silver concentration both in sample A and standard S was 107 ± 1 pg mL^−1^ and particle number concentration in the standard was 85,000 mL^−1^. Theoretically, if no changes of AgNPs occurred in sample A, the particle number concentration would be conserved. However, the particle number concentration decreased from 85,000 mL^−1^ to 64,000 mL^−1^, which represents a 25% drop. The reduction in both quantities (particle size and particle number) means that a significant portion of silver originally present in particles was dissolved and passed into the solution as silver ions (or smaller nanoparticles that cannot be detected by sp-ICP-MS). The dissolved silver portion was calculated by mass balance as 61 ± 5 pg mL^−1^, which represents 57% of the original mass. 

Digestion experiments with nanoparticles without the food matrix (sample B) showed different results (Figure 3). There is a slight decrease in the number of nanoparticles, from 85,000 to 70,000 mL^−1^, and a minor shift in the most abundant size of nanoparticles from 60 nm to 57 nm. Also, a minor population of 20 nm particles was observed. The mass balance showed that from 107 pg mL^−1^ of AgNPs only 74 pg mL^−1^ was conserved in nanoparticle state; in other words, 33 ± 4 pg mL^−1^ of silver was dissolved. 

To be able to differentiate the real changes in the nanoparticle system from the effect of the digested sample matrix on the results of sp-ICP-MS measurement, we repeatedly analysed the prepared control sample C. The comparison of AgNPs size distribution in control sample and standard is shown in Figure 5. Very similar patterns indicate that the effects of the digested sample matrix on the sp ICP-MS analysis of sufficiently diluted samples are minor or even negligible. The histograms of sample C and standard S overlap each other by 87%. The noticeable occurrence of small nanoparticles (in a diameter range from 18 to 30 nm) in control sample C suggests that at least some parts of the fractions that appeared in samples A and B are probably artefacts. 

## 4. Discussion

The extent of the AgNPs changes during digestion depends on the chemical environment of the nanoparticles resulting from composition of food digested together with the nanoparticles. Due to the chemical properties of silver [22], an oxidizing power is necessary to dissolve the silver particles (Ag(s) ⇌ Ag^+^(aq) + e^−^). The standard reduction potential for the redox equilibrium of Ag^+^/Ag (but taken a chemical reduction, i.e., in the inversed order) is as high as +0.80 V. Therefore, silver is resistant to oxidation. On the other hand, in strongly oxidising acidic conditions (e.g., in the presence of nitric acid) AgNPs are dissolved quickly. 

The main oxidising agent causing silver dissolution during simulated food digestion is oxygen, dissolved in the liquid phase of the sample via sample shaking during incubation. One could presume that the presence of the food matrix that contains reducing agents (sugars, phenolic compounds, ascorbic acid) might act as a stabiliser, preventing silver dissolution via oxygen consumption. However, the opposite is true. 

The comparison of digestion effects on the AgNPs treated with and without the bread sample matrix (Figure 4) shows that the food matrix causes the larger extent of changes, i.e., more silver is dissolved, and smaller nanoparticles remain in the digestate. This effect can be explained by silver ions chelating with the products of food enzyme hydrolysis (namely amino acids). The formation of silver complexes or chelates will shift the above mentioned redox equilibrium towards Ag^+^ ions. This explanation is also supported by the lower values of standard redox potential in the presence of ligands (e.g., in the presence of ammonia that forms [Ag(NH_3_)_2_]^+^ complex cations the with Ag^+^ ions, the standard potential is only +0.37 V [23]). In other words, in the presence of amino acids that can form silver complexes similar to those of ammonia, the oxidation of metallic silver is thermodynamically more favoured. Another phenomenon contributing to the larger extent of AgNPs changes could be the adsorption of Ag^+^ ions to some fractions of dietary fibre, present in the bread sample digestate as an insoluble residue.

As far as our results of AgNPs size distribution are concerned, they can be compared with those of two other studies [24,25]. In both, AgNPs standards were submitted to simulated digestion. While Walczak et al. [24] also conducted their experiments with 60 nm AgNPs standard and without a food matrix, Ramos et al. [25] performed the digestion of a chicken meat paste spiked with 40 nm AgNPs standard. The methodology of nanoparticles characterisation included sp-ICP-MS [24,25], dynamic light scattering [24] and scanning electron microscopy with energy-dispersive X-ray analysis [24]. The digestion procedure that these authors used was more complex, it also included salivary step. In both studies, a significant percentage of silver was conserved in the nanoparticle state; these findings roughly correspond to our results, but the portions of nanoparticles conserved or dissolved were different (see Table 4). 

The differences shown in Table 4 are likely caused by different conditions of experiments (smaller AgNPs used in [25] are less stable and tend more to dissolution).

## 5. Conclusions

Our experiments show that a significant part of AgNPs remains in the food sample even after simulated digestions. In the case of digestion of bread spiked with 59 nm AgNPs, a majority (about 60%) of the total silver mass is transferred to a dissolved form of silver and the most abundant nanoparticles diameter is decreased to 49 nm. The treatment of AgNPs themselves (without food matrix) causes moderate changes only. 

The analysis of the control sample showed that the results of sp-ICP-MS analyses are not affected by the food matrix if the samples are diluted enough (to achieve the final concentration of organic matter on a dry weight basis of 20 µg mL^−1^).

At least a part of AgNPs that appeared in food as a contaminant persists through the process of food digestion. Therefore, a portion of AgNPs ingested with food could penetrate the inner organs of the human body.

## Figures and Tables

**Table 1:** ICP-MS instrument settings.

Parameter	Setting for Conventional ICP-MS	Setting for sp-ICP-MS
Nebuliser gas flow	0.68–0.74 L min^−1^ *^a^*	*ca* 0.86 L min^−1^ *^b^*
Number of sweeps	20	1,200,000
Number of replicates	10	1
Isotopes (dwell time)	^107^Ag, ^109^Ag, ^103^Rh (20 ms for all)	^107^Ag (0.1 ms)
Peristaltic pump speed	48 min^−1^ (for accelerated prime of the new solution) 20 min^−1^ (for flow stabilisation and measurement)	constant speed of 17 min^−1^ (*ca* 0.32 mL min^−1^, the exact value was always determined)
Transport efficiency	not determined	*ca* 0.03–0.04 *^c^*
Data acquisition time	60.7 s	120 s

*^a^*—optimised for acceptable *I*(^140^Ce^16^O)/*I*(^140^Ce) ratio, i.e., <0.02. *^b^*—optimised for maximum sensitivity for ^107^Ag. *^c^*—always determined at the beginning of the measurement.

**Table 2:** The results of characterisation of two AgNPs standard dispersions.

Dispersion	*c*_Ag_ [fg mL^−1^]	*m*_average particle_ [fg]	*N* [mL^−1^]
AgNPs 59 ± 5 nm	21.47 × 10^9^	1.2601	1.70 × 10^10^
AgNPs 97 ± 11 nm	23.79 × 10^9^	5.4753	4.35 × 10^9^

**Table 3:** Design of the experiments.

Step	S	A	C	B	BB
sample weighing	–	✔	✔	–	–
+Ag NPs	–	✔	–	✔	–
+HCl + enzymes, simulated digestion	–	✔	✔	✔	✔
centrifugation	–	✔	✔	✔	✔
supernatant + gelatin	–	✔	✔	✔	✔
+Ag NPs	✔	–	✔	–	–
+gelatin	✔	–	–	–	–
final dilution and sp-ICPMS analysis	✔	✔	✔	✔	✔
*N*_teor_ [mL^−1^]	85,000	85,000	85,000	85,000	0

The symbol ✔ means that the corresponding operation step was done.

**Table 4:** Comparison of this study with those of Walczak et al. [24] and Ramos et al. [25].

	This Paper	Walczak et al. [24]	Ramos et al. [25]
Kind of AgNPs	59 nm AgNPs	60 nm AgNPs	40 nm AgNPs
Performance of digestion	two-step	three-step	three-step
Food matrix	wheat bread	no matrix	without matrix and with chicken meat paste
State of AgNPs in final digestate	minor drop of *N* (without matrix) significant drop of *N* and *d* (with bread)	minor drop of *N* (without matrix)	significant drop of *N* (without matrix) AgNPs (34–60 nm) are present (chicken)
Percentage of dissolved Ag	*ca* 30 (without matrix) *ca* 60 (with bread)	not determined	*ca* 67 (without matrix) *ca* 77 (with chicken)

## Data Availability

The original contributions presented in the study are included in the article, further inquiries can be directed to the corresponding author.
